# Evaluation of aortic valve stenosis from Phase-Contrast Magnetic Resonance data using a new automated segmentation and analysis method: Comparison against Doppler Echocardiography

**DOI:** 10.1186/1532-429X-13-S1-O30

**Published:** 2011-02-02

**Authors:** Carine Defrance, Emilie Bollache, Nadjia Kachenoura, Eric Bruguière, Alban Redheuil, Benoit Diebold, Ludivine Perdrix, Elie Mousseaux

**Affiliations:** 1 Hopital Européen Georges Pompidou, Paris, France; 2 INSERM U678, Paris, France

## Background

Aortic valve stenosis (AVS) is the most common valvular disease. Its evaluation is of growing interest because of its increasing incidence with the aging population. Previous studies demonstrated the usefulness of Phase-Contrast Magnetic Resonance (PCMR) images in the evaluation of AVS. However, because of the lack of automated methods for PCMR data analysis, this technique remains time-consuming and operator-dependent.

## Objectives

Therefore, the aims of this study were 1) to develop a semi-automated method for aortic flow analysis from PCMR images, and 2) to evaluate several approaches of aortic valve area (AVA) estimation.

## Methods

**Figure 1:**
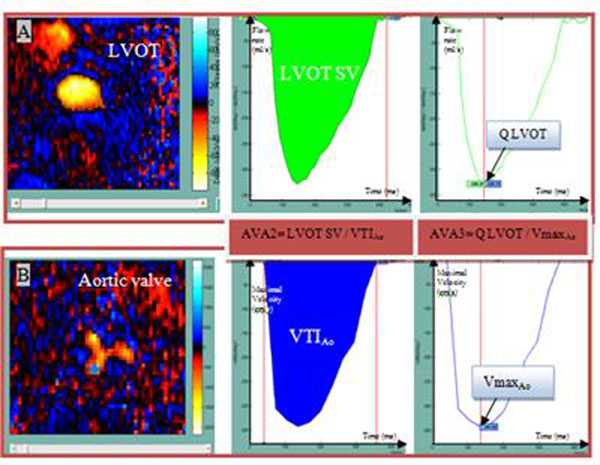
A) Example of left ventricular outflow tract (LVOT) segmentation on a velocity PC MR image, flow rate curve, whose integral corresponds to LVOT stroke volume (LVOT SV) and whose peak corresponds to Q LVOT Q; B) Example of aortic flow segmentation on a velocity PC MR image, transaortic maximal velocity curve, whose integral corresponds to VTI_AO_ and whose peak corresponds to Vmax_AO_. AVA2 is the ratio between the two integrals. AVA3 is the ratio between the two peaks.

We studied 37 consecutive patients with AVS (mean AVA:0,89 +/-0,42 cm^2^) and 12 healthy subjects (mean AVA: 3,19 +/- 0,65 cm^2^) who had the same day a trans-thoracic echocardiography (TTE) and PCMR acquisitions at the levels of the aortic valve and the left ventricle outflow tract (LVOT). PCMR data analysis included a semi-automated segmentation, based on pixels connectivity in terms of velocity sign, to delineate the aortic flow on all systolic frames, as well as a functional parameters extraction from aortic velocity and flow rate curves such as aortic maximal velocity (Vmax_Ao_) and AVA. AVA was calculated using: 1) Hakki’s formula which is a simplification of Gorlin’s formula, resulting in AVA1= cardiac output divided by √systolic pressure gradient, 2) the continuity equation with the most pertinent method found in previous studies in PCMR resulting in AVA2 = LVOT stroke volume (LVOT SV) divided by aortic valve velocity time integral (VTI_Ao_), and 3) the continuity equation with another approach previously described in echocardiography but never used in PCMR. It resulted in AVA3= LVOT peak Flow rate (QLVOT) divided by Vmax_Ao_ (figure 1).

## Results

The LVOT and aortic flows were successfully delineated on all phases for all subjects. Comparison of PCMR and echocardiographic Vmax_Ao_ resulted in a good correlation(r = 0.92). Hakki’s formula underestimated the AVA with regard to TTE in the absence of severe stenosis. AVA3 provided the best results (Table 1) in terms of detection of severe stenosis (r = 0.97; Specificity = 100% and Sensibility = 97%). Moreover, excellent intra-observer reproducibility was found (AVA3: ICC > 0.99 and mean differences: 0.00 ± 0.02 cm^2^; Vmax_Ao:_ ICC > 0.99 and mean differences: 0.02 ± 0.10 m/s).

**Table 1:** Comparison between TTE and PC MRI using the three methods for AVA estimation (*p<0.05)

	Mean (SD) of differences compared to TTE (cm^2^)	Correlation Coefficient R	Intra-class coefficient between TTE and PC MR	Specificity to detect severe stenosis (%)	Sensibility to detect severe stenosis (%)
AVA1	-0,50 (0,57)*	0,92	0 ,67	94	97
AVA2	+0,02 (0,37)	0,94	0,94	100	97
AVA3	+0,07 (0,29)	0,97	0,96	100	97

## Conclusion

Our semi-automated approach for AVS evaluation from PCMR provided reproducible velocity measurements and AVA estimates in good agreement with echocardiographic values, and were able to characterize the severity of AVS.

